# Predicting clinical benefit from everolimus in patients with advanced solid tumors, the CPCT-03 study

**DOI:** 10.18632/oncotarget.16029

**Published:** 2017-03-08

**Authors:** Fleur Weeber, Geert A. Cirkel, Marlous Hoogstraat, Sander Bins, Christa G.M. Gadellaa-van Hooijdonk, Salo Ooft, Erik van Werkhoven, Stefan M. Willems, Marijn van Stralen, Wouter B. Veldhuis, Nicolle J.M. Besselink, Hugo M. Horlings, Neeltje Steeghs, Maja J. de Jonge, Marlies H.G. Langenberg, Lodewyk F.A. Wessels, Edwin P.J.G. Cuppen, J.H. Schellens, Stefan Sleijfer, Martijn P. Lolkema, Emile E. Voest

**Affiliations:** ^1^ Center for Personalized Cancer Treatment, The Netherlands; ^2^ Department of Molecular Oncology, The Netherlands Cancer Institute, Amsterdam, The Netherlands; ^3^ Department of Medical Oncology, UMC Utrecht Cancer Center, Utrecht, The Netherlands; ^4^ Department of Molecular Carcinogenesis, The Netherlands Cancer Institute, Amsterdam, The Netherlands; ^5^ Department of Medical Oncology, Erasmus MC Cancer Institute, Rotterdam, The Netherlands; ^6^ Department of Radiotherapy, University Medical Center Utrecht, Utrecht, The Netherlands; ^7^ Department of Biometrics, The Netherlands Cancer Institute, Amsterdam, The Netherlands; ^8^ Department of Pathology, UMC Utrecht Cancer Center, Utrecht, The Netherlands; ^9^ Center for Image Sciences, University Medical Center Utrecht, Utrecht, The Netherlands; ^10^ Department of Radiology, UMC Utrecht Cancer Center, Utrecht, The Netherlands; ^11^ Department of Genetics, UMC Utrecht Center for Molecular Medicine, Utrecht, The Netherlands; ^12^ Department of Pathology, The Netherlands Cancer Institute, Amsterdam, The Netherlands; ^13^ Department of Medical Oncology, The Netherlands Cancer Institute, Amsterdam, The Netherlands; ^14^ Department of Clinical Pharmacology, The Netherlands Cancer Institute, Amsterdam, The Netherlands; ^15^ Faculty of EEMCS, Delft University of Technology, Delft, The Netherlands; ^16^ Cancer GenomiCs.nl, Utrecht, The Netherlands; ^17^ Hubrecht institute, Utrecht, The Netherlands; ^18^ Utrecht Institute of Pharmaceutical Sciences, Utrecht University, Utrecht, The Netherlands

**Keywords:** everolimus, biomarkers, predict response, time to progression ratio, clinical study

## Abstract

**Background:**

In this study, our aim was to identify molecular aberrations predictive for response to everolimus, an mTOR inhibitor, regardless of tumor type.

**Methods:**

To generate hypotheses about potential markers for sensitivity to mTOR inhibition, drug sensitivity and genomic profiles of 835 cell lines were analyzed. Subsequently, a multicenter study was conducted. Patients with advanced solid tumors lacking standard of care treatment options were included and underwent a pre-treatment tumor biopsy to enable DNA sequencing of 1,977 genes, derive copy number profiles and determine activation status of pS6 and pERK. Treatment benefit was determined according to TTP ratio and RECIST. We tested for associations between treatment benefit and single molecular aberrations, clusters of aberrations and pathway perturbation.

**Results:**

Cell line screens indicated several genes, such as *PTEN* (*P* = 0.016; Wald test), to be associated with sensitivity to mTOR inhibition. Subsequently 73 patients were included, of which 59 started treatment with everolimus. Response and molecular data were available from 43 patients. *PTEN* aberrations, i.e. copy number loss or mutation, were associated with treatment benefit (*P* = 0.046; Fisher's exact test).

**Conclusion:**

Loss-of-function aberrations in *PTEN* potentially represent a tumor type agnostic biomarker for benefit from everolimus and warrants further confirmation in subsequent studies.

## INTRODUCTION

The introduction of targeted therapy has been accompanied by an intensive search for biomarkers to select patients for treatment. The identification of companion diagnostics may improve treatment outcomes and cost-effectiveness of increasingly expensive oncolytic drugs. Several powerful biomarker-drug combinations have been introduced in the clinic, such as crizotinib in *ALK* mutant lung cancer and vemurafenib in *BRAF* V600E mutant melanoma [1, 2]. For these treatments, evident tumor regression can be observed in selected populations. However, for drugs that induce disease stabilization, such as everolimus, it is more difficult to determine treatment benefit. Everolimus is an orally administered drug with proven efficacy in advanced clear cell renal cell carcinoma, neuroendocrine tumors and breast cancer [3–6]. Everolimus inhibits the mammalian Target of Rapamycin (mTOR) pathway and its downstream substrates, S6K and 4EBP1, which promote cell growth, proliferation and survival [7]. mTOR can be activated by upstream pathways such as the MAPK pathway and the AKT/PI3K pathway [7]. Previous studies have described several genetic aberrations that might be predictive for response to mTOR inhibition such as mutations in or loss of *PIK3CA*, *PTEN, TSC* and *KRAS* [8–22].

These genetic aberrations in the mTOR pathway and its interconnected pathways are present across many different tumor types (COSMIC database). It is therefore reasonable to believe that patients with other tumor types harboring the proper molecular profile might also benefit from treatment [21, 23]. Unfortunately, despite extensive knowledge on the mechanism of action of everolimus, no tissue broad biomarkers have yet been identified and clinically validated.

To address this issue, we revised genomic profiles and drug sensitivity of 835 cell lines to generate hypothesis about potential biomarkers, and conducted a prospective biomarker identification study for everolimus. This study was conducted by the Center for Personalized Cancer Treatment (www.cpct.nl), a large consortium of hospitals in the Netherlands devoted to personalized medicine.

## RESULTS

### Exploration of cell line data

The GDSC1000 cell line data from the Sanger Institute was used to search for potential markers for treatment sensitivity. As everolimus was not screened, we used the rapamycin analog temsirolimus as a proxy. IC50 values for temsirolimus were available for 835 cell lines, and sensitivity differed significantly between tumor types (*p* < 0.001; ANOVA). Specifically, non-small cell lung cancer (NSCLC), neuroblastoma, pancreatic and colon tumor cell lines were in general more resistant than e.g. kidney and bladder tumors (Supplementary Figure 1). After selecting only solid tumors and correcting for tissue of origin, the elastic net analysis identified a small number of genetic aberrations that could be associated with response: *PTEN* mutations, *FGFR2* mutations and *CDKN2A* loss were associated with increased sensitivity (Table 1). Gains in *CCNE1* and *ERCC5*, as well as mutations in *RB1, HGF, SOX9* and *CIC* were associated with temsirolimus resistance (Table 1). The strongest effect was seen for *CCNE1* gain and *FGFR2* mutations. Gain of *CCNE1* was observed in 48 cell lines including breast (10/42) and NSCLC (11/100). Only within breast cancer cell lines, was *CCNE1* gain alone also associated with temsirolimus resistance (*P* = 0.010; one-tailed *t*-test). *FGFR2* mutations were only observed in eight cell lines, distributed over seven tumor types.

**Table 1:** Cell line data

genetic aberration	Estimate	Std Error	*t*-value	Pr(>|t|)	sign level
FGFR2_mut	-2,014	0,505	-3,989	0,000	***
CCNE1 gain	0,897	0,242	3,714	0,000	***
PTEN_mut	-0,536	0,192	-2,788	0,005	**
CDKN2A loss	-0,307	0,116	-2,645	0,008	**
RB1_mut	0,486	0,198	2,447	0,015	*
CIC_mut	1,324	0,550	2,408	0,016	*
gain_cnaPANCAN384_,ERCC5,ING1,IRS2,TFDP1,	0,523	0,223	2,346	0,019	*
SRGAP3 loss	0,328	0,141	2,334	0,020	*
loss_cnaPANCAN216	0,724	0,313	2,313	0,021	*
HGF_mut	1,408	0,700	2,011	0,045	*
SOX9_mut	1,048	0,544	1,927	0,054	.
**genetic aberration**	**Estimate**	**Std Error**	***t*-value**	**Pr(>|t|)**	**sign level**
PTEN_mut	-0,457	0,189	-2,422	0,016	*
PIK3CA_mut	-0,384	0,188	-2,044	0,041	*
gain_cnaPANCAN164_,KRAS,	0,285	0,171	1,660	0,097	.
gain_cnaPANCAN395_,AKT1,HSP90AA1,PPP2R5C,	0,411	0,316	1,298	0,195	
loss_cnaPANCAN44_,BMPR1A,FAS,PTEN,	-0,208	0,183	-1,137	0,256	
KRAS_mut	-0,193	0,195	-0,985	0,325	
TSC1_mut	0,307	0,490	0,627	0,531	
gain_cnaPANCAN129_,MET,	0,092	0,195	0,470	0,639	
EGFR_mut	-0,037	0,340	-0,110	0,912	
gain_cnaPANCAN301_,CDK12,ERBB2,MED24,	-0,018	0,246	-0,071	0,943	
gain_cnaPANCAN124_,EGFR,	0,010	0,180	0,055	0,956	

Legend: This table illustrates genetic aberrations that could potentially predict sensitivity to mTOR inhibition, based on an in vitro drug screen with temsirolimus. Part A of the table demonstrates the relation between genetic aberrations and sensitivity to temsirolimus, corrected for tumor type and excluding blood cell tumors. Part B shows similar data, but is analyzed per gene and specifically directed at genetic aberrations previously associated with sensitivity to mTOR inhibition.

Next, we focused on genes in the mTOR pathway or genes previously reported in association with sensitivity to mTOR inhibition. In our model including all tumor types, both *PIK3CA* and *PTEN* mutations were associated with increased sensitivity (*P* = 0.041 and *P* = 0.016, respectively; Wald test) (Table 1). *PIK3CA* mutations were most common in breast, NSCLC, ovarian, stomach, colorectal and aerodigestive tract tumor cell lines. *PTEN* mutations were also frequently observed in endometrial tumors. Within those subtypes, *PIK3CA* mutations were only associated with temsirolimus sensitivity in tumor cells from the aerodigestive tract (*P* = 0.014; one-tailed *t*-test) and cervical tumor cell lines (*P* = 0.023), but no such association could be seen for *PIK3CA* mutation status in breast cancer (*P* = 0.411), and an opposite effect was observed in endometrial cancer (*P* = 0.053). *PTEN* mutations were significantly associated with response in ovarian (*P* = 0.0211) and endometrial tumor cells (*P* = 0.031), but not in breast cancer or colorectal cancer cell lines (*P* = 0.278 and *P* = 0.423, respectively).

### Patient baseline data

**Figure 1:**
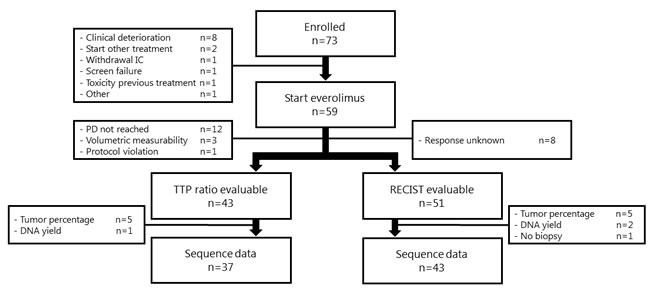
Evaluability of patients This figure illustrates the evaluability of patients for the biomarker analyses. A single patient can be evaluable according to both RECIST and TTP ratio. Abbreviations: IC, Informed Consent; PD, Progressive Disease.

A total of 73 patients were included in the study. Seventy-one (97%) patients underwent a tumor biopsy according to protocol. Fifty-nine patients (81%) started treatment with everolimus, of whom 43 (59%) were evaluable for efficacy according to TTP ratio and 51 (70%) for efficacy according to RECIST. Tumor material sufficient for sequencing analysis was obtained in 37 patients (51%) of the TTP cohort and 43 patients (59%) of the RECIST cohort (Figure 1). Nine biopsies were insufficient for sequencing due to a low or absent tumor percentage. Six biopsies were insufficient due to a low DNA yield. We obtained sequence data of 1,977 genes for 38 patients, and whole exome sequencing data for five patients. The sequencing data reached an average coverage of 159x. All 43 patients were also sequenced on the IonTorrent (panel of 50 genes and custom-made primers for mTOR pathway related genes) to validate the mutations. Baseline characteristics of the patients are described in Table 2.

**Figure 2:**
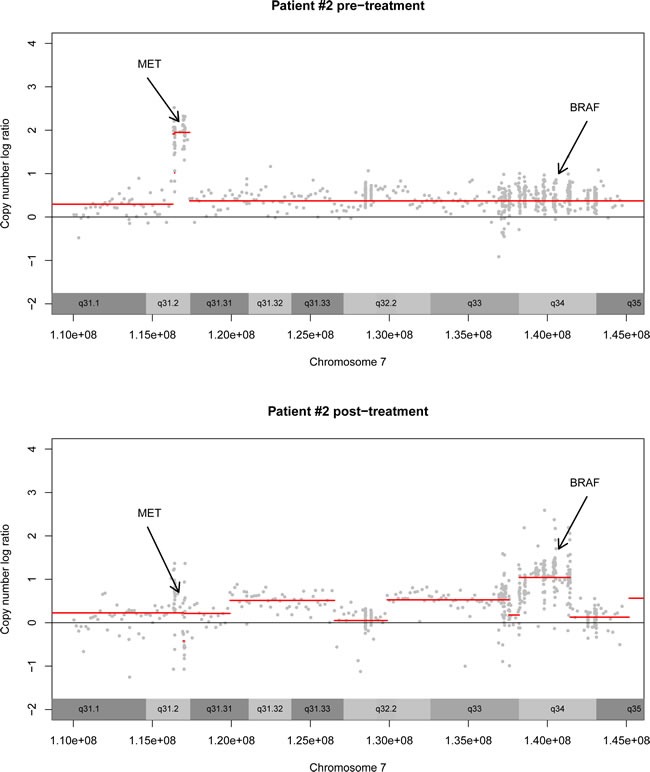
Pre-post treatment biopsy This figure demonstrates the copy number profile of chromosome 7 in patient #2 pre-treatment and post-treatment. Pre-treatment, there is an amplification of *MET*. This amplification is not present in the post-treatment biopsy. Instead, there is an amplification of BRAF wild-type.

**Table 2:** Baseline characteristics

Demographic or Clinical Characteristic	No. of patients	%
**No. of patients**	43	
**Sex** Male	26	60.5
**Age, years** Mean Range	60 31 – 79	
**WHO PS** 0 1 2 Missing	14 26 1 2	32.6 60.5 2.3 4.7
**Primary tumor** Colorectal NET Esophageal Breast NSCLC Ovarian Renal cell Sarcoma Cervical Head and Neck Bladder Mesothelioma Thyroid Thymoma Gastric Pancreatic Melanoma Unknown origin	12 7 4 3 2 2 2 1 1 1 1 1 1 1 1 1 1 1	27.9 16.3 9.3 7.0 4.7 4.7 4.7 2.3 2.3 2.3 2.3 2.3 2.3 2.3 2.3 2.3 2.3 2.3
**No. of previous treatments** 1 2 3 >3	9 5 4 25	20.9 11.6 9.3 58.2
**Biopsy characteristics** Tumor percentage - Median - SD DNA yield (ng) - Median - SD	60 23 1440 2123	

Legend: This table contains the baseline characteristics of all patients of whom both molecular and clinical response data was available. DNA yield is depicted in nanogram.

### Tumor mutation and copy number data

We detected on average sixteen somatic mutations per patient in the 1,977 gene set (range zero to eighty-six). The most frequently mutated genes included *TP53* (*N* = 24), *APC* (*N* = 9), *KRAS* (*N* = 9) and *PIK3CA* (*N* = 7). In addition, we detected copy number gains and amplification of several well-known oncogenes such as *ERBB2* (*N* = 6), *PIK3CA* (*N* = 4), *CCND1* (*N* = 3), *MYC* (*N* = 3), *EGFR* (*N* = 2), *MET* (*N* = 2), *MDM2* (*N* = 2) and *KRAS* (*N* = 1), and amplification of *TERT* in 5 samples. Losses were detected of *SMAD4* and *CDKN2A* (both *N* = 8), *TP53* (*N* = 5), *APC* (*N* = 5), *PTEN, VHL* and *RB1* (all *N* = 4), and specifically *TSC1* (*N* = 3) and *TSC2* (*N* = 1).

### Genomic variations and treatment response in patients

When exploring the cell line data, several hypotheses were generated with regard to the correlation between genomic aberrations and treatment response. The first step was to evaluate if these hypotheses could be tested in our patient data set. The rest of the paragraph is focused on TTP ratio assessment, because only one patient had a RECIST response, and because PFS is a longitudinal endpoint similar to TTP ratio, but without the correction for individual tumor growth rate.

*In vitro* data suggested increased resistance to mTOR inhibitors in the presence of a gain of *CCNE1* or mutation in *RB1*. In our patient data however, all patients with a gain of *CCNE1* (*N* = 2) or mutation in *RB1* (*N* = 2) had clinical benefit from treatment (defined as TTP ratio response) (Table 3, Supplementary Table 1). Mutations in *FGFR2*, *PTEN* and loss of *CDKN2A* were associated with increased sensitivity to mTOR inhibition *in vitro*. In our patient data set, there was only one patient with a loss of *FGFR2*, this patient had a favorable outcome in terms of TTP ratio. Loss of *CDKN2A* (*N* = 7) was not correlated with TTP ratio response (either as a binary outcome or as a continuous outcome). Five patients had either a mutation or copy number loss of *PTEN*. Despite the fact that it was only possible to generate a TTP ratio for three of these patients (which classified them as responders) the other two patients also had clinical signs of a treatment effect: in one patient, central necrosis of all target lesions was observed at first response evaluation whereas the other patient had a PFS of 90 weeks, with a TTP1 period of 23 weeks. This patient was not evaluable for TTP ratio analysis, due to loss of volumetric measurability. When categorizing these five patients as responders, there was a significant correlation between treatment response and *PTEN* status (*P* = 0.046; one-tailed Fisher's exact test). It should be noted however that these *PTEN* aberrations often coincided with other mTOR pathway related mutations (Supplementary Table 1). *PIK3CA* was also associated with increased sensitivity to mTOR inhibition *in vitro*. However, we could not find an association in our patient data (seven responders *versus* three non-responders).

**Table 3:** Genetic aberrations and response

Gene	Clinical benefit		Statistics
	Yes	No	*p*
*KRAS*	5	5	.327
*PIK3CA*	7	3	.377
*MAPK*	5	4	.623
*CDKN2A*	5	2	.326
*PTEN*	5	0	.046*
*ERBB2*	3	2	.625
*TSC1*	2	2	NA
*AKT*	2	0	NA
*CCNE1*	2	0	NA
*RB1*	2	0	NA
*TSC2*	1	0	NA
*MTOR*	1	0	NA
*FGFR2*	1	0	NA

Legend: This table contains the number of patients that have, or have not experienced clinical benefit from treatment, stratified per afflicted gene.

Using an unbiased, overall analysis, no other somatic mutations or copy number alterations showed a significant correlation with response. Similarly, combining genetic aberrations or comparing somatic mutations on the pathway level did not yield significant results. To evaluate if genetic aberrations had a downstream effect by activating respectively the mTOR or MAPK pathway, we evaluated pS6 and pERK status. However, when incorporating pS6 and pERK status in previously mentioned analyses, we were still not able to predict clinical benefit, nor were pS6 and pERK predictive for response as single markers.

When focusing on mTOR (or interconnected) pathway related genes, we observed an equal distribution of responders and non-responders in *KRAS* mutated patients. This equal distribution was also observed in patients with other MAPK mutated genes. More directly upstream of *MTOR* are *TSC1* and *TSC2*. One breast cancer patient harbored a missense *TSC1* mutation and indeed responded to everolimus. Four tumor samples showed copy number loss or a mutation of *TSC1* and one tumor showed loss of *TSC2*; these events were evenly divided over responders and non-responders.

### Pre- and post-treatment comparison

Nine patients underwent a post-treatment biopsy procedure, of which four biopsies were of sufficient quality for DNA sequencing. Two of these patients had a TTP ratio response. In patient #1 (breast cancer), no resistance mechanisms were detected. Patient #2's tumor initially harbored a very focal, high level amplification of the *MET* proto-oncogene (Figure 2). During treatment this amplification was clearly reduced, while a second high level gain on chromosome 7 appeared, i.e. affecting *BRAF*.

## DISCUSSION

In this study, we found that copy number loss or mutation of *PTEN* was associated with treatment benefit of everolimus, suggesting that *PTEN* status could be a predictive biomarker for benefit from treatment. *PTEN* was frequently speculated to be a marker of interest, however, most clinical biomarker studies did not find a significant correlation with response [8, 9, 11, 12, 14, 17, 18]. This could be a result of the method used to determine *PTEN* status, as many studies used immunohistochemistry instead of DNA sequencing. Janku et al. were one of the few to combine immunohistochemistry and DNA sequencing in their biomarker study and had similar findings to our study [14]. Furthermore, this was the first study to employ an intra-patient control to determine treatment benefit. These findings should be further confirmed in other trial designs, such as basket studies.

Although currently published (pre-)clinical data report contradictory results, *KRAS* and *PIK3CA* mutation status have previously been associated with respectively resistance and sensitivity to mTOR inhibition in particular tumor types. In our dataset, we did not observe an association between mutations in either of these genes and treatment response. However, the sample size of this study is insufficient to make any statements regarding the absence of such an association.

Interestingly, a new amplification of wild-type *BRAF* was identified in a post-treatment biopsy, suggestive of a potential mechanism of resistance to mTOR inhibition. This tumor had a pre-treatment *MET* amplification. Both *MET* and *BRAF* can activate the MAPK-signaling pathway, but while *MET* functions upstream of mTOR, *BRAF* is just downstream of mTOR/Akt so activation of MAPK at this level circumvents the possible effect of mTOR inhibition. This data illustrates, that although post-treatment biopsies are difficult to acquire, they do provide hypothesis-generating information.

While this study yielded interesting findings and the data produced will be released to large sequencing databases to facilitate data sharing in further biomarker discovery efforts, there is an important side note. This study was drafted and implemented five years ago, when next generation sequencing technology had only just found its way to research centers and hospitals worldwide. The unprecedented wealth of genetic information fueled faith and optimism to identify markers for response and select patients for treatment. The past years have revealed that the implementation of genomics-based personalized medicine is not as simple as initially thought [24]. Complicating factors are amongst others varying degrees of tumor type dependence for the efficacy of biomarker-drug combinations, discrepancies between *in vitro* and in-patient findings, and a lower than expected incidence of actionable mutations [24]. In our study, the discrepancies between *in vitro* and in-patient findings can also be a result of the use of different mTOR inhibitors. Negative results for the first large genotype-matched drug trial (SHIVA), where context, i.c. tumor type, dependence was not taken into account, have also raised concerns [24, 25]. Another major hurdle is, that for many targeted agents, there are no established biomarkers. To identify single (or combinations of) molecular alterations that can predict treatment outcome, other study designs with more homogeneous patient groups (basket trials) or large cohorts of patients ( > 1000) are necessary [26, 27]. The latter can only be achieved by world-wide collaborations and sharing of data [28]. National- and worldwide sequencing initiatives such as the CPCT or project GENIE (by the American Association of Cancer Research) have been established to facilitate these efforts [28]. And whereas many of these efforts mainly focus on genomics-based analyses, we should aim to incorporate other types of analyses such as transcriptomics or proteomics.

To conclude, this study identified an association between *PTEN* status and treatment benefit from everolimus, identifying PTEN status as a potential biomarker for everolimus therapy. *BRAF* wildtype amplification could be a potential mechanism of resistance.

## MATERIALS AND METHODS

### Cell line data

Genetic profiles and drug sensitivity measurements of cell lines treated with the mTOR inhibitor temsirolimus were analyzed for potential biomarkers for treatment. This dataset (GDSC1000 v17a) was downloaded from http://cancerrxgene.org/gdsc1000/Pharmacogenomic_interactions.html [29].

### Patients

The CPCT-03 study was an open-label, single arm, biomarker study. The primary objective was to identify genetic predictors for response to mTOR inhibition by everolimus. Patients with advanced solid malignancies without regular treatment options were eligible for inclusion. Inclusion and exclusion criteria, as well as detailed information on everolimus treatment, safety assessments and study design have been described previously [30]. The protocol was approved by the Institutional Review Board of The Netherlands Cancer Institute and complied with the Declaration of Helsinki, Dutch law and Good Clinical Practice guidelines. All patients provided written informed consent prior to study-related procedures. Patients were accrued at the Netherlands Cancer Institute, UMC Utrecht Cancer Center, and Erasmus MC Cancer Institute. The study was registered on ClinicalTrials.gov (NCT01566279).

### Clinical efficacy assessments

Efficacy was measured according to three endpoints, TTP ratio, Response Rate (RR) and Progression-free survival (PFS). The TTP ratio uses an intra-patient control to correct for natural tumor growth rate and has been described previously by Cirkel et al. [30].

### Tumor biopsy

After inclusion, all patients underwent a pre-treatment histological tumor biopsy of a metastatic lesion. A post-treatment tumor biopsy was optional. Biopsies were snap-frozen and stored at -80°C. Safety and feasibility of the CPCT ‘biopsy pipeline’ has been described by Bins et al. [31]. Blood samples (10mL) were collected in K2EDTA tubes, as a reference to determine somatic mutations.

### Evaluability

Patients evaluable according to either RECIST or TTP ratio were evaluable for biomarker analyses in case of an adequate tumor biopsy (tumor percentage ≥30% and DNA yield ≥500ng).

### DNA sequencing

Histological assessment to confirm the presence of tumor cells and mark regions with high tumor cellularity for macro-dissection was performed by a pathologist (S.W.). DNA was extracted from whole blood and macro-dissected tumor sections. Barcoded libraries were generated as previously described and enriched for a “Cancer mini-genome” of 1,977 cancer genes, based on Vermaat et al. and Hoogstraat et al. [32–34]. Enriched libraries were sequenced to an average coverage of 150x on a SOLiD 5500xl instrument according to manufacturer's protocol. Whole exome sequencing was performed for six patients using the NextSeq 500 v2 as our sequencing facility switched platforms. Somatic mutations were validated with the Ion Ampliseq Cancer Panel or custom-made primers for mTOR-related genes. Mapping, variant calling and annotation was done as previously described [33]. Sam tools mpileup was used to ensure the absence or presence of a variant in a given sample [35]. Copy number profiles were generated using CNVkit [36]. Detailed information on sequencing methods and bioinformatics pipelines can be reviewed in online-only supplementary materials.

### Immunohistochemistry

In order to determine activation of mTOR and interconnected pathways, all available biopsies (*N* = 33) were stained for phospho-S6 and phospho-ERK. Phospho-S6 is a marker for activation of mTOR, pERK is a marker for MAPK pathway activation. Slides were scored for intensity (0-3) and percentage of positive tumor cells by a pathologist blinded for treatment outcome.

### Statistical analyses

No formal sample size calculation was performed due to an unknown expected RR of a heterogeneous group of tumors with unknown frequencies of genetic aberrations that might be predictive for response. The study was open for accrual of 60 evaluable patients or 15 evaluable TTP ratio responders. R (version 3.2.1) was used for downstream analyses of mutations and copy number variation, and to detect associations between genetic variation, tumor type and treatment response. All genetic aberrations (copy number gain, loss or mutation) were encoded as binary variables, where 0 = absence and 1 = presence of the mutation. On the cell line data, elastic net feature selection was performed using the R-package glmnet [37]. We used linear models to assess the significance of the presence of (multiple) genetic aberrations while correcting for tumor type and ANOVA to determine the effect of tissue type on treatment response. Univariate analyses of single genes within specified tumor types were done using one- or two-tailed *t*-tests, depending on context. If previous data or literature had already provided an indication of the direction of the effect, a one-tailed test was used. In our patient data, we tested associations between single variables and response using Fisher's exact test and associations between multiple variables and outcome were modeled using logistic regression. We assessed pathway enrichment of genetic variation in responders and non-responders as described previously [38]. Briefly, we used the Kyoto Encyclopedia of Genes and Genomes (KEGG) to define pathways. A pathway was considered to be affected if at least one of its genes was found mutated. We performed the Fisher's exact test to correlate pathway activation and treatment response.

## SUPPLEMENTARY MATERIALS FIGURES





## Abbreviations

4EBP1: 4E-Binding Protein
APC: Adenomatous Polyposis Coli
AKT: Protein kinase B
ALK: Anaplastic Lymphoma Kinase
ANOVA: Analysis of Variance
BRAF: B-Raf Proto-Oncogene, Serine/Threonine Kinase
CDKN2A: Cyclin-Dependent Kinase Inhibitor 2A
CCND1: Cyclin-D1
CCNE1: Cyclin-E1
CIC: Capicua transcriptional repressor
COSMIC: Catalogue Of Somatic Mutations In Cancer
CPCT: Center for Personalized Cancer Treatment
DNA: Deoxyribonucleic acid
EGFR: Epidermal Growth Factor Receptor
ERBB2: Erb-B2 Receptor Tyrosine Kinase 2
ERCC5: Excision Repair Cross Complement group 5
FGFR2: Fibroblast Growth Factor Receptor-2
HGF: Hepatocyte Growth Factor
IC50: Inhibitory Concentration Of 50%
KEGG: Kyoto Encyclopedia of Genes and Genomes
KRAS: Kirsten rat sarcoma viral oncogene homolog
MAPK: Mitogen-Activated Protein Kinases
MDM2: Mouse double minute 2 homolog
MET: proto-oncogene MET
mTOR: mammalian Target of Rapamycin
MYC: Avian Myelocytomatosis Viral Oncogene Homolog
NSCLC: Non-Small Cell Lung Cancer
pERK: phospho Extracellular Regulated Kinase
PFS: Progression-Free Survival
PI3K/PIK3CA: Phosphoinositide-3-kinase
pS6: phosphorylated S6 Ribosomal Protein
PTEN: Phosphatase and tensin homolog
SOX9: SRY-Box 9
RB1: Retinoblastoma 1
RECIST: Response Evaluation Criteria In Solid Tumors
RR: Response Rate
TERT: Telomerase Reverse Transcriptase
TP53: Tumor Protein p53
TSC: Tuberous Sclerosis Complex
TTP: Time To Progression
VHL: Von Hippel-Lindau.
