# Supplementary material for: Predicting clinical benefit from everolimus in patients with advanced solid tumors, the CPCT-03 study
Source: Oncotarget. 2017 Mar 8;8(33):55582–92. doi: 10.18632/oncotarget.16029 (PMC5589684; doi:10.18632/oncotarget.16029)
Supplement: Supplementary file 1 [file oncotarget-08-55582-s001.pdf]

Predicting clinical benefit from everolimus in patients with advanced solid tumors, the CPCT-03 study

Supplementary Material

DNA SEQUENCING

DNA was extracted from 500µl whole blood and from 20µm macro-dissected tumor sections using NorDiag Arrow (Isogen Life Science, De Meern, the Netherlands). DNA was quantified with Qubit 2.0 fluorometer® (Life Technologies, Carlsbad CA, USA).

Single nucleotide variants (SNVs) and small insertions/deletions (indels) were detected in a designed "Cancer mini-genome" consisting of 1,977 cancer genes, based on Vermaat et al. and Hoogstraat et al. [1, 2]. Barcoded fragment libraries were generated from 500ng DNA from tumor and control blood samples as previously described [3]. Pools of libraries were enriched for this gene set using SureSelect technology (Agilent Technologies, Santa Clara California, USA). Enriched libraries were sequenced to an average coverage of 150x on a SOLiD 5500xl instrument according to manufacturer's protocol.

Whole exome sequencing was performed on samples from six patients using the NextSeq 500 v2 by Illumina (San Diego CA, USA) as our sequencing facility switched to another sequencing platform. Barcoded libraries were derived using the KAPA DNA library kit (KAPA Biosystems, London, UK). Barcoded adaptors were from NEXTflex-96™DNA. Barcoded adaptors by Bioo Scientific (Austin TX, USA). Samples were enriched whole exome using the SureSelect technology All Exon V5 kit (Agilent Technologies, Santa Clara California, USA) and sequenced to an average coverage of 75x (reference) and 150x (tumor). Mapping, variant calling and annotation was done as previously described [1]. Somatic variants were extracted by comparing variant lists of both tumor and control samples and subsequently genotyping discordant positions in the raw datasets of all three samples using sam tools mpileup to ensure the absence or presence of the variant in a given sample [4]. Copy number profiles based on the targeted sequencing data were generated using CNVkit [5].

All samples were also analyzed using the Ion Ampliseq Cancer Panel (Thermo Fisher Scientific, Waltham MA, USA) to validate findings from SOLiD and Illumina sequencing and were processed according to manufacturer's protocol. Each sample was barcoded using the Ion Express barcoded adapters (Thermo Fisher Scientific, Waltham MA, USA) allowing multiplexed sequencing. A total of 16 PCR cycles were performed on fresh frozen samples, plus an additional 5 cycles at the end of the library prep to obtain sufficient DNA for sequencing. Libraries were quantified using the Qubit fluorometer (Life Technologies, Carlsbad CA, USA), pooled, and diluted to a concentration of 0.00104ng/µl, which was further processed for sequencing using the Ion OneTouch (Life Technologies, Carlsbad CA, USA). Samples were sequenced on a 318v2 chip, allowing the simultaneous sequencing of fourteen samples per run, aiming for a minimum average coverage of 1000x per sample. Base calling and alignment of the IonTorrent sequencing data were performed using the standard Ion Torrent software (Torrent suite 4.0). Mutations in mTOR pathway related genes that were not included in the Ion Ampliseq Cancer Panel were validated using custom-made primers (pcr fragments between 100-200 basepairs). Library prep and barcoding was done using the KAPA high throughput Library prep kit (KAPA Biosystems: KK8234) without the a-tailing reaction. For each sample 2.5µl universal and 2.5µl barcoded IonTorrent adapors was used and the libraries were amplified using 5µl primermix and 7 PCR cycles.

Immunohistochemistry

In order to determine activation of mTOR and interconnected pathways, all available biopsies (*N*=33) were stained for phospho-S6 (Phospho-S6 Ribosomal (Ser240/244) by Cell Signaling Technologies, Danvers MA, USA) and phospho-ERK (Phospho-P44/42 (Erk1/2) (Thr202/Tyr204) by Cell Signaling Technologies, Danvers MA, USA). Phospho-S6 is a marker for activation of mTOR, pERK is a marker for MAPK pathway activation. All samples were converted to FFPE, immunohistochemistry was performed on a BenchMark Ultra autostainer (Ventana Medical Systems, Tuscon AZ, USA). Briefly, paraffin sections were cut at 3 um, heated at 75°C for 28 minutes and deparaffinized in the instrument with EZ prep solution (Ventana Medical Systems, Tuscon AZ, USA). Heat-induced antigen retrieval was carried out using Cell Conditioning 1 (Ventana Medical Systems, Tuscon AZ, USA) for 32 minutes at 95^{0}C (Phospho-S6 Ribosomal (Ser240/244)), or 64 minutes at 95^{0}C (Phospho-P44/42 (Erk1/2) (Thr202/Tyr204)). Phospho-P44/42 (Erk1/2) (Thr202/Tyr204) was detected using

clone D13.14.4E (1/400 dilution, 1 hour 36°C, Cell Signaling Technologies, Danvers MA, USA), Phospho-S6 Ribosomal (Ser240/244) was detected using clone D68F8 (1/1000 dilution, 32 minutes at 36°C, Cell Signaling Technologies, Danvers MA, USA). Bound antibody was detected using the UltraView DAB Detection kit (Phospho-P44/42 (Erk1/2) (Thr202/Tyr204)) or the OptiView DAB Detection Kit (Phospho-S6 Ribosomal (Ser240/244)) (Ventana Medical Systems, Tuscon AZ, USA). Slides were counterstained with Hematoxylin II and Bluing Reagent (Ventana Medical Systems, Tuscon AZ, USA). Slides were scored for intensity (0-3) and percentage of positive tumor cells by a pathologist blinded for treatment outcome.

REFERENCE LIST

1. Hoogstraat M, de Pagter MS, Cirkel GA, van Roosmalen MJ, Harkins TT, Duran K, Kreeftmeijer J, Renkens I, Witteveen PO, Lee CC, Nijman IJ, Guy T, van 't Slot R, et al. Genomic and transcriptomic plasticity in treatment-naive ovarian cancer. Genome Res. 2014; 24: 200-211.
2. Vermaat JS, Nijman IJ, Koudijs MJ, Gerritse FL, Scherer SJ, Mokry M, Roessingh WM, Lansu N, de Bruijn E, van Hillegersberg R, van Diest PJ, Cuppen E, Voest EE. Primary colorectal cancers and their subsequent hepatic metastases are genetically different: implications for selection of patients for targeted treatment. Clin Cancer Res. 2012; 18: 688-699.
3. Harakalova M, Mokry M, Hrdlickova B, Renkens I, Duran K, van Roekel H, Lansu N, van Roosmalen M, de Bruijn E, Nijman IJ, Kloosterman WP, Cuppen E. Multiplexed array-based and in-solution genomic enrichment for flexible and cost-effective targeted next-generation sequencing. Nat Protoc. 2011; 6: 1870-1886.
4. Li H. A statistical framework for SNP calling, mutation discovery, association mapping and population genetical parameter estimation from sequencing data. Bioinformatics. 2011; 27: 2987-2993.
5. Talevich E, Shain AH, Botton T, Bastian BC. CNVkit: Genome-Wide Copy Number Detection and Visualization from Targeted DNA Sequencing. PLoS Comput Biol. 2016; 12: e1004873.

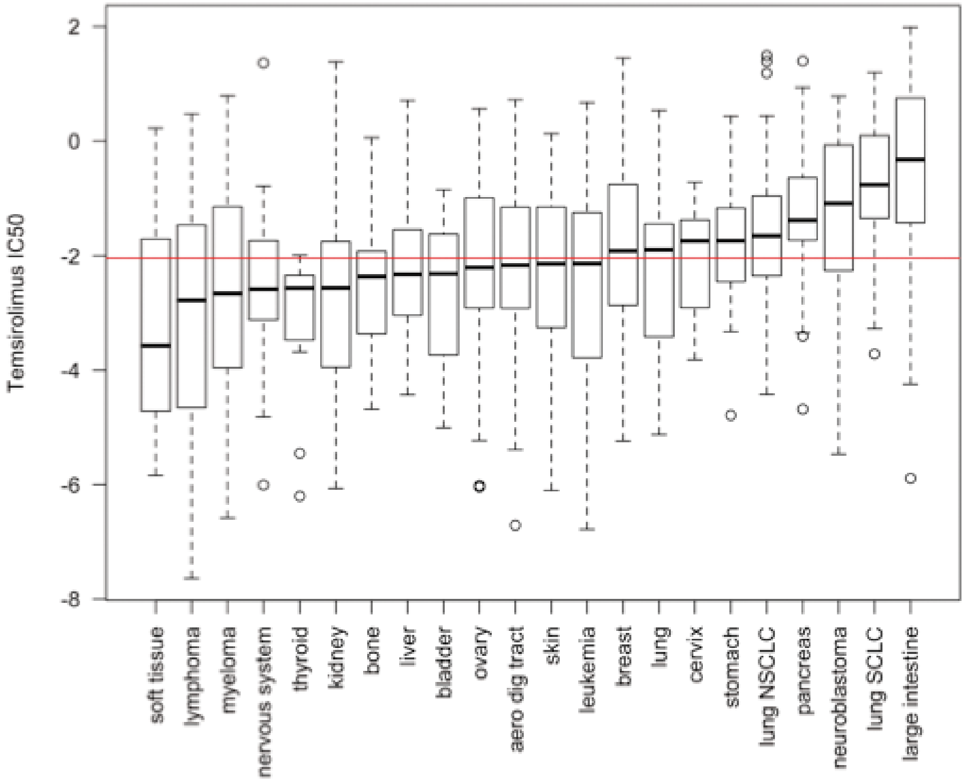

Supplementary Figure 1: Tumor type and *in vitro* response
